# Facile Synthesis of Layer Structured GeP_3_/C with Stable Chemical Bonding for Enhanced Lithium-Ion Storage

**DOI:** 10.1038/srep43582

**Published:** 2017-02-27

**Authors:** Wen Qi, Haihua Zhao, Ying Wu, Hong Zeng, Tao Tao, Chao Chen, Chunjiang Kuang, Shaoxiong Zhou, Yunhui Huang

**Affiliations:** 1 Beijing Key Laboratory of Energy Nanomaterials, Advanced Technology & Materials Co., Ltd, China Iron & steel Research Institute Group, Beijing 100081, P.R. China; 2 State Key Laboratory of Material Processing and Die & Mould Technology, School of Materials Science and Engineering, Huazhong University of Science and Technology, Wuhan, Hubei 430074, P.R. China; 3 School of Materials and Energy, Guangdong University of Technology, Guangzhou, 510006, P.R. China

## Abstract

Recently, metal phosphides have been investigated as potential anode materials because of higher specific capacity compared with those of carbonaceous materials. However, the rapid capacity fade upon cycling leads to poor durability and short cycle life, which cannot meet the need of lithium-ion batteries with high energy density. Herein, we report a layer-structured GeP_3_/C nanocomposite anode material with high performance prepared by a facial and large-scale ball milling method *via in-situ* mechanical reaction. The P-O-C bonds are formed in the composite, leading to close contact between GeP_3_ and carbon. As a result, the GeP_3_/C anode displays excellent lithium storage performance with a high reversible capacity up to 1109 mA h g^−1^ after 130 cycles at a current density of 0.1 A g^−1^. Even at high current densities of 2 and 5 A g^−1^, the reversible capacities are still as high as 590 and 425 mA h g^−1^, respectively. This suggests that the GeP_3_/C composite is promising to achieve high-energy lithium-ion batteries and the mechanical milling is an efficient method to fabricate such composite electrode materials especially for large-scale application.

Li-ion batteries (LIBs) have been extensively used to power portable electronics and electric vehicles because of high energy density and long cycle life. In order to meet the need for LIBs with high energy and low cost, it is essential to develop large-capacity electrodes made from nontoxic, low cost, and abundant materials^1^,^2^,^3^,^4^,^5^,^6^,^7^,^8^,^9^. Group IVA elements (Si, Ge, Sn etc.) based alloys with high theoretical capacities have been reported as potential anode materials. Recently, Ge has attracted more and more attention due to large gravimetric capacity (1624 mA h g^−1^), good lithium diffusion, high electrical conductivity and great oxidation resistance^10^,^11^,^12^,^13^,^14^,^15^,^16^,^17^,^18^. However, Ge suffers dramatic volumetric change (270%) during Li alloying/de-alloying process, which leads to the pulverization of particles, destabilization of solid electrolyte interphase (SEI) films and hence poor cyclability^19^,^20^,^21^.

To overcome the fast capacity fade of Ge, various Ge-based alloys have been designed. For example, inactive metal was used as host matrix to accommodate the large volumetric change^12^,^22^, ^23^,^23^. In addition, nanocrystallization is an effective strategy for Ge-based alloys to avoid the pulverization during cycling, such as fabricating tailored morphology^10^,^18^,^19^, hollow structure^14^,^15^,^17^, nanoparticles^24^ and carbon-based composites^25^. Several recent studies have illustrated that phosphorus could serve as volume buffer material in metal phosphides and exhibit improved lithium ion storage and sodium ion storage during alloy process^26^,^27^,^28^,^29^,^30^,^31^,^32^,^33^,^34^. Cui’s^35^ and Wang’s groups^36^,^37^ found stable P-C and P-O-C bonding in the phosphorus-based composites and obtained high capacity and excellent rate capability even after extended cycles. Manthiram *et al*. embedded CuP_2_ nanoparticles into the carbon matrix to get improved electrochemical performance^30^. It is suggested that the formed stable P-O-C can increase the contact between active materials, accommodate large volume change and preserve mechanical integrity during cycling. Zhou *et al*. reported that GeP_5_/C exhibited a specific capacity as high as 2300 mA h g^−1^ that could be maintained to 40 cycles^38^. However, the long-term cycling stability caused by the huge volume expansion and poor interaction during cycling is still a challenge for practical application. Although the nanostructured materials have been investigated to significant improve electrochemical performances for high capacity anode, it is still limited for commercial applications due to the complex synthesis procedures^9^. The facile synthesis of nanomaterials is needed to reduce the cost for large-scale application.

Here, we develop a simple and large scale method to prepare nanostructured GeP_3_/C composite in which layer structured GeP_3_ nanoparticles are *in-situ* formed and embedded by carbon layer. The carbon not only works as conducting matrix but also facilitates to form stable P-O-C bonding with GeP_3_ to accommodate the volume change. The as-obtained GeP_3_/C composite exhibits a high reversible capacity of 1109 mA h g^−1^, good cyclability with 86% retention over 130 cycles, and excellent rate capability, which could be promising as anode material for LIBs with high energy density.

## Results

**Figure 1:**
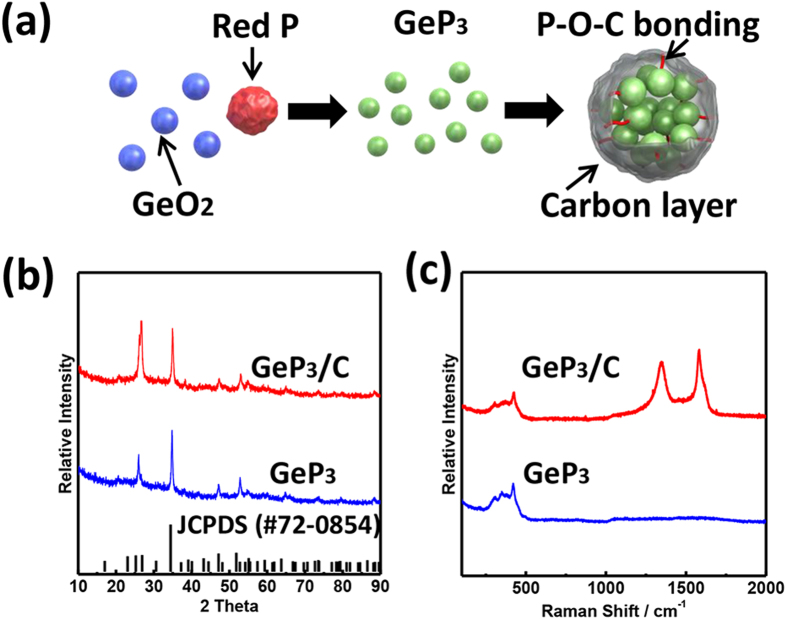
Synthesis and characterization of GeP_3_/C: (**a**) schematic illustration of ball milling process from red P, GeO_2_ and carbon; (**b**) XRD patterns of GeP_3_ and GeP_3_/C; (**c**) Raman spectra of GeP_3_ and GeP_3_/C.

The GeP_3_/C composite was synthesized *via* HEMM method with GeO_2_ powder, red P and carbon as starting materials. The HEMM can not only provide enough energy to make the phase change *via* mechanical reaction, but also peel apart the layered materials by shear force. With HEMM, the red P reacts with GeO_2_ to form GeP_3_ phase, and the GeP_3_ particles are further turned to small ones. Meanwhile, carbon is coated onto GeP_3_ particles *via* ball milling to enhance the conductivity. Thus the nanostructured GeP_3_/C composite is attained, as illustrated in Fig. 1a. The reaction can be expressed as below in which the extra oxygen is absorbed on the surface of GeP_3_:


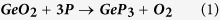


The phase purity and structure of GeP_3_ and GeP_3_/C were checked by XRD and Raman spectra. After first-step ball milling, the diffractions are well indexed to pure GeP_3_ phase (JCPDS No. 72–0854) with rhombohedral crystal structure, which is similar to layer structured GeP_5_ with good conductivity^38^,^39^. After second-step ball milling together with carbon, the peak intensity at 34° corresponding to (202) plane for GeP_3_ decreases, indicative of further refinement of particles. For comparison, if we directly ball mill red P, GeO_2_ and carbon at same condition, only GeO_2_ diffraction peaks appear (see Supplementary Fig. S1), demonstrating that GeP_3_ phase cannot be formed by such one-step ball milling process. This is because carbon prevents GeP_3_ from reacting with GeO_2_.

The structure of the composite was further detected by Raman spectra (Fig. 1c). The broad peak in the region of 300–500 cm^−1^ can be defined to GeP_3_. After carbon coating, the typical D and G band are observed to indicate the disorder carbon caused by mechanical impact and shear force during the ball-milling process. Our previous studies^40^,^41^ on the ball milled graphitic materials show that the higher intensity of D band (corresponding to the disordered C-C bond) would benefit the lithium storage. In this work, we prolonged ball milling time and obtained increased intensity of D band (*I*_D_/*I*_G_ = 0.84) in the GeP_3_/C composite, demonstrating that the average size of the sp^2^ domains decreases because of the mechanical shear exfoliation of carbon in the HEMM.

**Figure 2:**
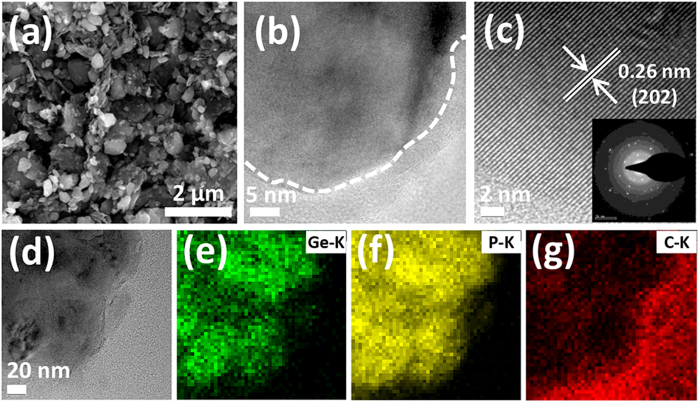
(**a**) SEM image of GeP_3_/C; (**b**) typical TEM image of GeP_3_/C; (**c**) HRTEM image of GeP_3_/C, the inset is the selected area electron diffraction (SAED); (**d**–**g**) TEM image and corresponding elemental mappings of Ge, P and C.

Figure 2 shows the morphology of GeP_3_/C composite. The average particle size is 200–300 nm (Fig. 2a), much smaller than those of starting red P, GeO_2_ and GeP_3_ particles (see Supplementary Figs S2–S4). To further confirm the actual composition of GeP_3_, the EDS spectrum as well as results are presented in Fig. S4b. The molar ratio of Ge/P is 1:3.05, close to the designed composition. The HRTEM image and the selected area electron diffraction (SAED) (Fig. 2b,c) clearly show that GeP_3_ nanoparticles surrounded by carbon layers have a basal distance of 0.26 nm, which is consistent with the (202) lattice spacing of GeP_3_ phase (JCPDS 72-0854). The SAED in the inset of Fig. 2c reveals the well crystallized GeP_3_. From the elemental mappings of GeP_3_/C in Fig. 2d–f, we can see the overlapped Ge signal and P signal, which further confirms the formation of GeP_3_ phase. The carbon layer coated on the GeP_3_ surface can be clearly observed. We can also see that GeP_3_ nanoparticles are well dispersed in the carbon matrix, which can facilitate the transfer of electrons and ions. The carbon layer acts not only as conductive network to enhance the conductivity, but also as mechanical buffer to accommodate large volume change during cycling. In addition, the surface area of GeP_3_ is 5.3 m^2^ g^−1^, while that of GeP_3_/C is 25.1 m^2^ g^−1^ (see Supplementary Fig. S5), indicating that the surface area is greatly enhanced due to the carbon coating.

**Figure 3:**
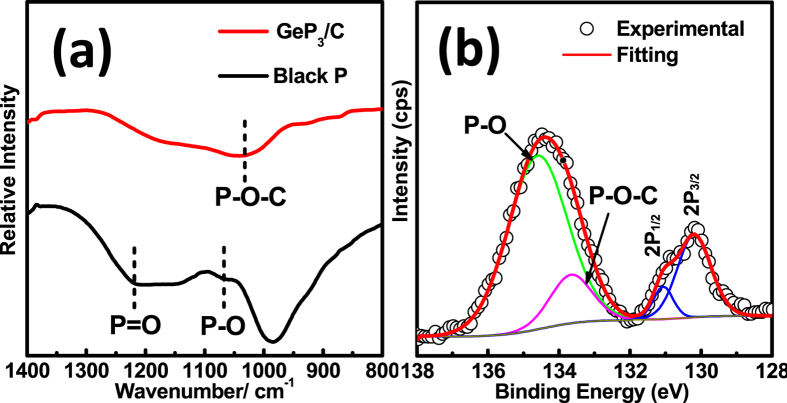
(**a**) FT-IR spectra of black P and GeP_3_/C; (**b**) high-resolution XPS P2p spectrum of GeP_3_/C.

The interaction between carbon and GeP_3_ particles was further investigated by FT-IR and XPS. The ball milled red P can be transferred to black P^37^,^42^, which makes it easy to absorb oxygen to form P-O and P=O bond signalled by the IR peak at around 1080 and 1200 cm^−1^, respectively. In the GeP_3_/C composite, the P-O and P=O peaks almost disappear but the additional P-O-C peak located at 1008 cm^−1^ is detected, which may be due to the residual oxygen formed by reduction reaction between GeO_2_ and red phosphorus during the HEMM process (Fig. 3a)^30^,^36^,^43^. The high-resolution P 2p XPS spectrum in Fig. 3b can be used to examine the surface electronic state and the formation of P-O-C bond. The peaks at 130.2 and 131.1 eV are ascribed to 2P_3/2_ and 2P_1/2_, respectively. The peak at 134 eV can be fitted into two peaks at 133.6 and 134.6 eV, corresponding to P-O-C and P-O bond^30^,^44^, agreeing well with the FT-IR observation. The P–O–C bond is expected to enable the carbon matrix to get strong chemical binding with the GeP_3_ particles, and hence to improve electrochemical reversibility and cycling stability for the GeP_3_/C composite electrode.

**Figure 4:**
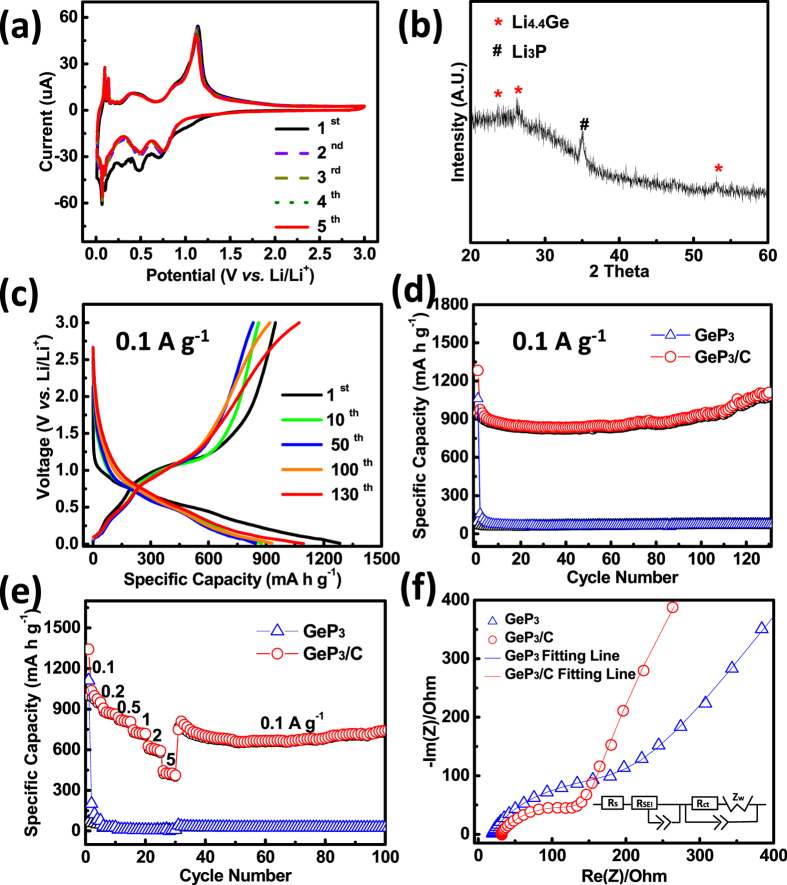
(**a**) CV curves of GeP_3_/C obtained at a scan rate of 0.01 mV s^−1^; (**b**) *ex-situ* XRD pattern of GeP_3_ after 1^st^ discharge at a current density of 0.01 A g^−1^; (**c**) selected charge/discharge curves of GeP_3_/C at 0.1 A g^−1^; (**d**) cycling performance and (**e**) rate capability of GeP_3_ and GeP_3_/C (the specific capacity is calculated based on the whole electrode); (**f**) EIS curves of GeP_3_ and GeP_3_/C before cycling. The inset is the equivalent circuit model for the simulation.

The electrochemical performances of GeP_3_/C composite are shown in Fig. 4. From the CV curves of GeP_3_/C in Fig. 4a, we can see two clear sharp cathodic peaks at 0.48 and 0.7 V during the first lithiation step, corresponding to the formation of Li_x_P^35^,^36^,^38^. The cathodic peak at ~0.2 V is believed to indicate the further alloying of Li^+^ with Ge to form Li_x_Ge. Two anodic peaks observed at 0.4 and 1.1 V are ascribed to the reversible reaction of Li_x_Ge and Li_x_P, respectively. For comparison, the CV curves of black P/C and GeO_2_/C confirm the above reactions (see Supplementary Figs S6 and S7). The cathodic and anodic peaks below 0.1 V are correlated with the insertion and desertion of Li^+^ ions into the carbon layer^41^. In the second cycle, the two main cathodic peaks slightly shift to 0.5 and 0.76 V due to polarization and the structural change of GeP_3_ after the Li-ion insertion in the first cycle. In the subsequent cycles, both the peak current and the integral area are almost overlapped, demonstrating the high capacity reversibility and good stability of GeP_3_/C.

To further investigate the lithium storage mechanism of the GeP_3_ composite, the *ex-situ* XRD was performed after first discharge to the voltage of 0.01 V (Fig. 4b). The peaks can be well assigned to Li_3_P and Li_4.4_Ge^38^,^45^. Combined with the CV curves and *ex-situ* XRD patterns, the lithium storage mechanism of GeP_3_ can be described as follows:


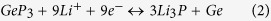



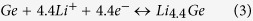



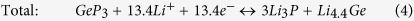


The calculated theoretical capacity of GeP_3_ is 1581 mA h g^−1^, in which P atoms contribute 1062 mA h g^−1^ and Ge atoms contribute another 519 mA h g^−1^. According to the TG curves (Fig. S8), the calculated carbon content in the composite is 25.4 wt.%, less than the designed one (30 wt.% carbon in the mixture), which can be ascribed to the partial carbonization during TG test. Therefore, the theoretical capacity of the GeP_3_/C composite should be 1218.3 mA h g^−1^, in which the contributions from GeP_3_ and carbon are 1106.7 and 111.6 mA h g^−1^, respectively.

The charge/discharge profiles of the half cell with GeP_3_/C composite as cathode from 1^st^ cycle to 130^th^ cycle are shown in Fig. 4c. The initial discharge and charge capacities are 1283 and 948 mA h g^−1^, respectively. The initial coulombic efficiency is 73.8%, which is mainly ascribed to the formation of an SEI layer on the electrode surface. Fig. 4d,e show the cyclability and rate capability for GeP_3_ and GeP_3_/C. For pure GeP_3_, the capacity fades rapidly within several cycles, which is due to the large volume change that causes pulverization of the active material. Interestingly, the HEMM-derived GeP_3_/C composite shows much better electrochemical performances. At a current density of 0.1 A g^−1^, a reversible capacity as high as 1109 mA h g^−1^ is attained, and the capacity retention is about 86% over 130 cycles, which can be attributed to the nanostructured electrodes for the improved Li-ion accessibility upon cycling process^41^. Even at high current densities of 2 and 5 A g^−1^, the GeP_3_/C still exhibits specific capacities of 590 and 425 mA h g^−1^, respectively. After running for 30 cycles at various current densities, the capacity of GeP_3_/C can be recovered to 900 mA h g^−1^ and maintained well during another 70 cycles when the current is tuned back to 0.1 A g^−1^, indicative of excellent rate capability and cyclability. The long-term cycling performance of GeP_3_/C at high current density of 1 A g^−1^ was also tested. As seen in Fig. S9, it still delivers stable specific capacity of 312 mA h g^−1^ after 1000 cycles.

To verify the improved performance of GeP_3_/C composite, EIS was used to compare GeP_3_ and GeP_3_/C. The Nyquist plots of both samples before cycling measured within a frequency range of 1000 kHz to 0.001 Hz are shown in Fig. 4f. The SEI resistance (*R*_SEI_) and the charge transfer resistance (*R*_ct_) are simulated by EC-Lab software with an equivalent circuit model (see the inset of Fig. 4f) that is fitted well with experimental data. The diameter of the semicircle is a measure of the *R*_ct_, which is related to the electrochemical reaction between the particles or between the electrode and the electrolyte. The diameter of the semi-circle for GeP_3_/C is smaller than that of GeP_3_, indicative of lower charge transfer resistance. In the low frequency region, the GeP_3_/C electrode exhibits a shortened and more inclined line with a higher slope compared with GeP_3_ electrode, demonstrating that GeP_3_/C exhibits faster Li^+^ diffusion. The results suggest that carbon coating *via* HEMM benefits the electron transformation between the electrode and the electrolyte.

Therefore, the excellent electrochemical performances of GeP_3_/C can be explained by at least two reasons. On one hand, the carbon layer can provide good electron transportation and hence enhance the electronic contact between the active particles. On the other hand, the stable P-O-C bonding between active materials and carbon matrix can alleviate the huge volumetric change upon Li^+^ interaction/extraction in GeP_3_. The *ex-situ* SEM images of GeP_3_/C electrode after 30 cycles at 0.1 A g^−1^ in Fig. S10 (see Supplementary) further confirm the stable microstructure of active materials during cycling. The top and cross sections of SEM images show smooth surface after cycling, suggesting the improved mechanical integrity of active materials. The TEM image along with elemental mappings after 30 cycles (see Supplementary Fig. S11) also indicate the structure stability over continuous expansion/contraction during cycling, in which P, Ge and C elements are uniformly distributed. This maintained morphology demonstrates the good conductive network and stable chemical bonding in GeP_3_/C, which is responsible from another point of view for the excellent electrochemical performance.

In summary, nanostructured GeP_3_/C composite has been designed and synthesized *via* a developed simple high-energy ball mill method with red phosphorus, GeO_2_ and graphite as starting materials. With conductive carbon as layer to form stable P-O-C chemical bonding, the GeP_3_/C composite exhibits a reversible capacity as high as 1109 mA h g^−1^ at 0.1 A g^−1^ with nearly 86% capacity retention over 130 cycles. Moreover, high rate capacity and stable cycling performance are also achieved. We believe that the current HEMM method is suitable for facile and large scale synthetic strategy to prepare nanostructured alloy anode materials, and that the HEMM-derived GeP_3_/C composite is promising as anode material for the next-generation lithium-ion batteries with high energy density.

## Methods

### Experimental

The GeP_3_/C composite was synthesized by two-step high energy mechanical milling (HEMM) method. Commercial red phosphorus (98%, Alfa Aesar), GeO_2_ (99%, Alfa Aesar) were mixed with a molar ratio of 3:1 in a stainless steel vial (250 mL) and sealed in an argon-filled glove box, followed by HEMM at 400 rpm for 40 h on P5 ball milling machine (Pritsch, Germany). The weight ratio of ball to powder was 20:1. The obtained powder (GeP_3_) was further mixed with carbon (99%, Alfa Aesar) in a weight ratio of 7:3 for another 50 h ball milling at 150 rpm to get the final product of GeP_3_/C nanocomposite. The sample for comparison was prepared by mixing red phosphorus, GeO_2_ and carbon with the same condition *via* one-step HEMM. The black phosphorus was prepared from red phosphorus by HEMM at 400 rpm for 40 h. The P/C was made by mixing black phosphorus and carbon in a weight ratio of 7:3 at 150 rpm for 50 h ball milling. The GeO_2_/C was also made with the same condition by mixing GeO_2_ and carbon.

### Characterization

The phase purity and crystal structure of the samples were examined by X-ray diffraction (XRD, Bruker D8, Germany) with Cu Kα radiation at 40 kV and 40 mA from 10° to 90°. Raman measurement was carried out on a HORIBA JOBIN YVON S.A.S. system (LabRAM HR800, Japan) at 532 nm. The X-ray photoelectron spectrometer (XPS) data were collected with an ESCALab220i-XL electron spectrometer from VG Scientific using 300 W Al K radiations. The binding energies obtained in the XPS analysis were corrected with reference to C1s (284.8 eV). Fourier transform infrared spectrometry (FTIR) was performed on Nicolet-6700 (Thermo Scientific, United State). The morphology, microstructure and corresponding energy-dispersive X-ray spectrometry (EDS) of samples were characterized by field-emission scanning electron microscope (SEM, FEI NANO 450, United State). High-resolution transmission electron microscopy (HRTEM), selected-area electron diffraction (SAED) and EDS mapping were carried out by a Tecnai G2 F20 operating at 200 kV. Thermogravimetry (TG) analysis was performed with a NETZSCH STA 449 C in the temperature range of 50–1200 °C at a heating rate of 10 °C min^−1^ in air. Nitrogen sorption isotherms were obtained using a Quantachrome Autosorb automated gas sorption system at −196 °C. Specific surface areas were calculated using the Brunauer-Emmett-Teller (BET) theory.

### Electrochemical Measurement

The 2025-type coin cells were used for electrochemical test. The working electrode was made by a coating technique. The active material, carbon black and polyvinylidene fluoride (70:10:20 by weight ratio) were completely mixed in N-methyl-2-pyrrolidone to achieve a slurry. The slurry was then coated onto copper foil. The electrodes were dried in a vacuum oven for 24 h before cell assembly. The loading mass of active material was about 1.3 mg cm^−2^. The coin cells were assembled in an argon filled glove box (Braun, Germany). Li metal foil was used as a counter electrode, and Celgard 2400 as the separator. The electrolyte was 1 mol L^−1^ LiPF_6_ in a mixed solvent of ethylene carbonate (EC), diethyl carbonate (DMC) and fluoroethylene carbonate (EMC) (1:1:1 by volume). Galvanostatic charge/discharge tests were carried out on a battery test system (Land BT2001A, Wuhan, China) between 0.01 and 3.0 V *versus* Li/Li^+^. Cyclic voltammetry (CV) was performed at a scan rate of 0.01 mV s^−1^ within the range of 0.01–3.0 V on an electrochemical workstation (VMP3, Bio-Logic SA, France). Electrochemical impedance spectroscopy (EIS) was measured by applying a sine wave with amplitude of 5 mV in the frequency range from 1000 kHz to 1 Hz.

## Additional Information

**How to cite this article**: Qi, W. *et al*. Facile Synthesis of Layer Structured GeP_3_/C with Stable Chemical Bonding for Enhanced Lithium-Ion Storage. *Sci. Rep.* **7**, 43582; doi: 10.1038/srep43582 (2017).

**Publisher's note:** Springer Nature remains neutral with regard to jurisdictional claims in published maps and institutional affiliations.

## Supplementary Material

Supplementary Information
