# Supplementary material for: Facile Synthesis of Layer Structured GeP3/C with Stable Chemical Bonding for Enhanced Lithium-Ion Storage
Source: Sci Rep. 2017 Feb 27;7:43582. doi: 10.1038/srep43582 (PMC5327472; doi:10.1038/srep43582)
Supplement: Supplementary Information [file srep43582-s1.pdf]

Facile Synthesis of Layer Structured GeP_3/C with Stable Chemical Bonding for Enhanced Lithium-Ion Storage

Wen Qi[1], Haihua Zhao[1,2], Ying Wu[1], Hong Zeng[1], Tao Tao[3], Chao Chen[3], Chunjiang Kuang[1], Shaoxiong Zhou[1]*, Yunhui Huang[2]*

[1] Beijing Key Laboratory of Energy Nanomaterials, Advanced Technology & Materials Co., Ltd, China Iron & steel Research Institute Group, Beijing 100081, P.R.China.
[2] State Key Laboratory of Material Processing and Die & Mould Technology, School of Materials Science and Engineering, Huazhong University of Science and Technology, Wuhan, Hubei 430074, P. R. China.
[3] School of Materials and Energy, Guangdong University of Technology, Guangzhou, 510006, P. R. China.

Correspondence and requests for materials should be addressed to Y.H.H. (huangyh@mail.hust.edu.cn) and S.X.Z. (sxzhou@atmcn.com).

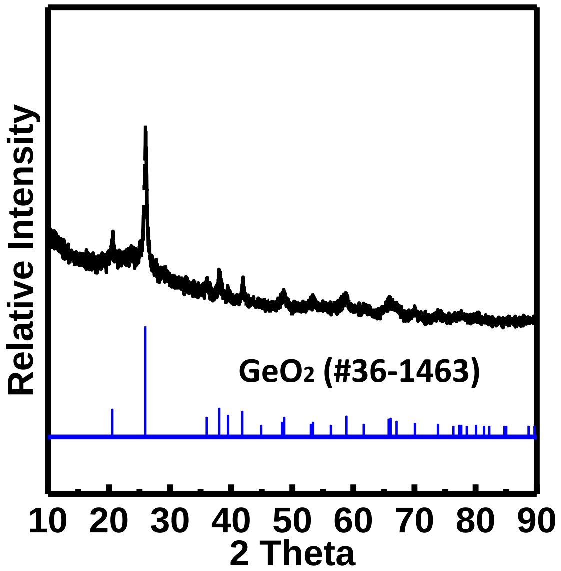

Fig. S1 XRD pattern of the GeP_3/C sample obtained by one-step ball milling of GeO_2, red P and carbon.

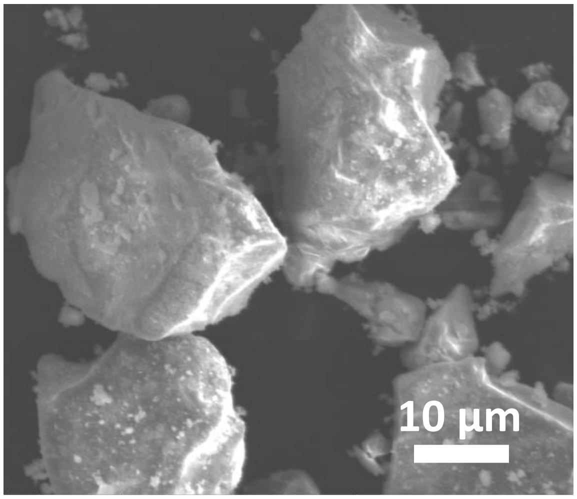

Fig. S2 SEM image of red P starting material.

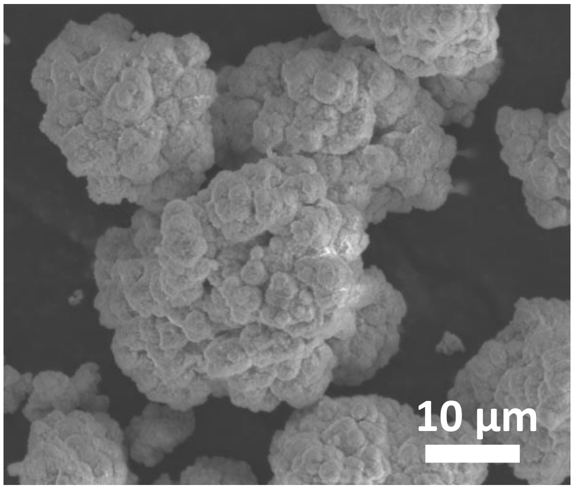

Fig. S3 SEM image of GeO_2 starting material.

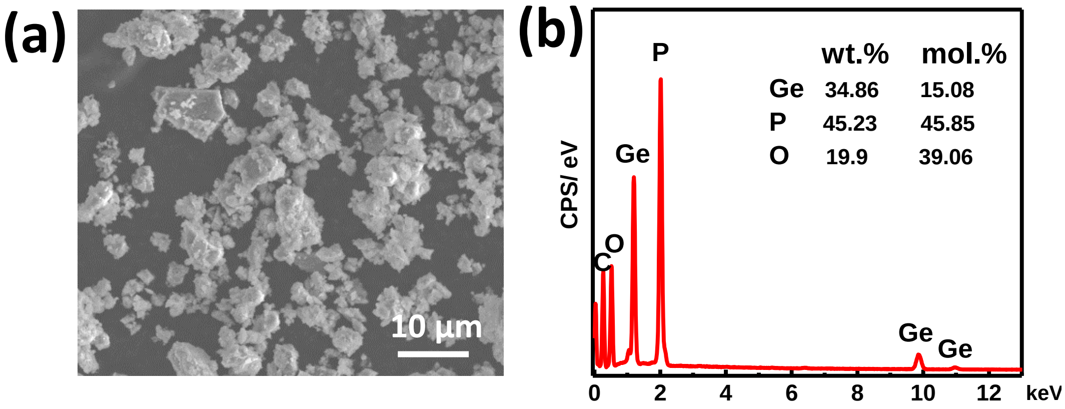

Fig. S4 a) SEM image of as-made GeP_3 and b) corresponding ESD spectrum.

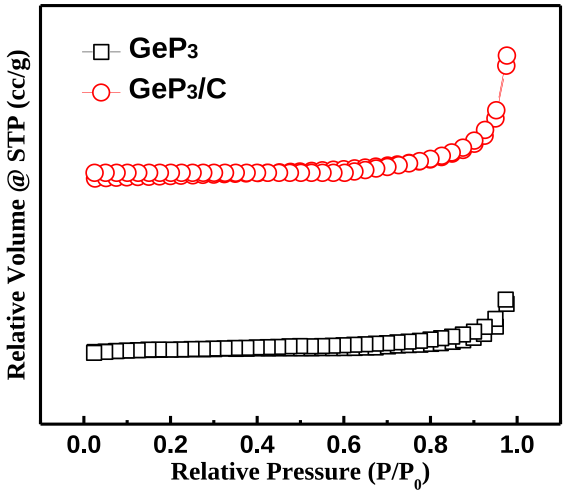

Fig. S5 Nitrogen isotherms of GeP_3 and GeP_3/C.

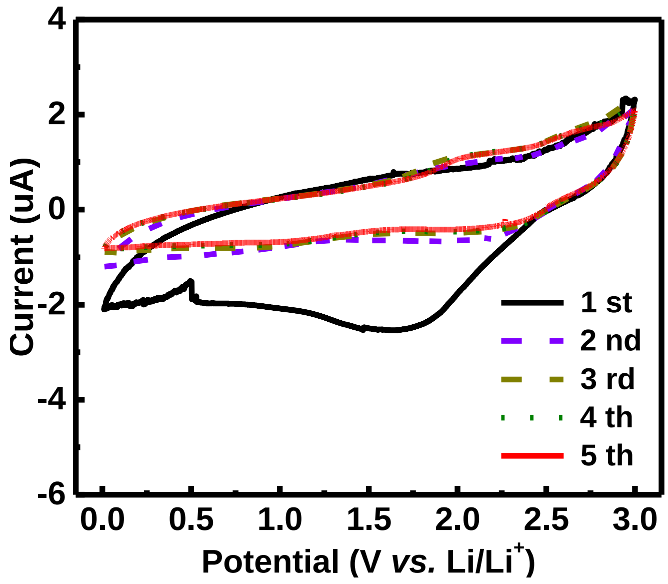

Fig. S6 CV curves of P/C obtained at a scan rate of 0.1 mV s^{-1}.

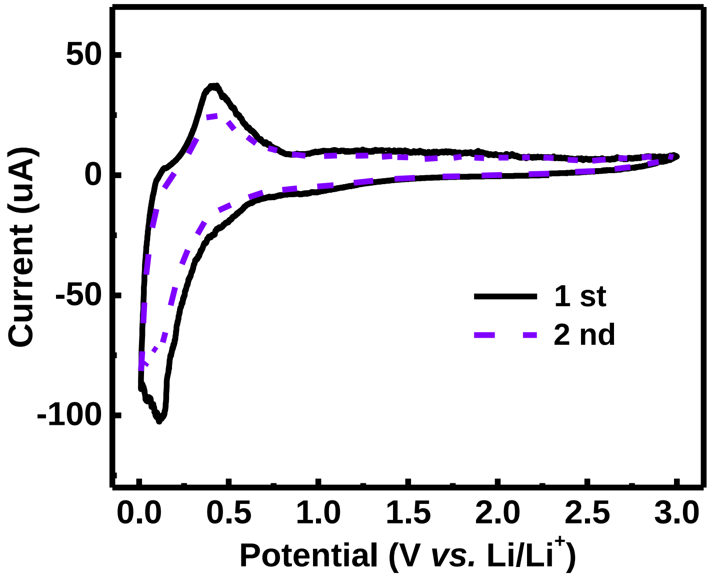

Fig. S7 CV curves of GeO_2/C obtained at a scan rate of 0.1 mV s^{-1}.

Thermogravimetric (TG) and differential scanning calorimetry (DSC) analysis were used to identify the mass contents of carbon in GeP_3/C composites (Figure S8). The initial weight loss (~ 1 wt.%) before 100 ℃ is attributed to the evaporation of water. The pure GeP_3 curve with weight gain (~ 7.4 wt. % at 1200 ℃) shows the endothermic peak at 530 ℃, which corresponds to the oxidization of GeP_3 in air atmosphere. As for GeP_3/C composites, the weight gain from 100 ℃ to 720 ℃ is also ascribed to the oxidization of GeP_3. From 720 to 820 ℃, there is a weak endothermic peak indicating the partially carbonization between carbon and GeP_3. Further increasing the heating temperature, the residual carbon in the mixture is oxidized to release CO_2 gas for the weight loss (18 wt.%). As consequence, the carbon content in GeP_3/C composite, estimated from the weight loss of GeP_3/C plus the weight gain from the oxidation of GeP_3, is ~ 25.4 wt.%. The mass loss of 4.6 wt.% can be ascribed to the partial carbonization.

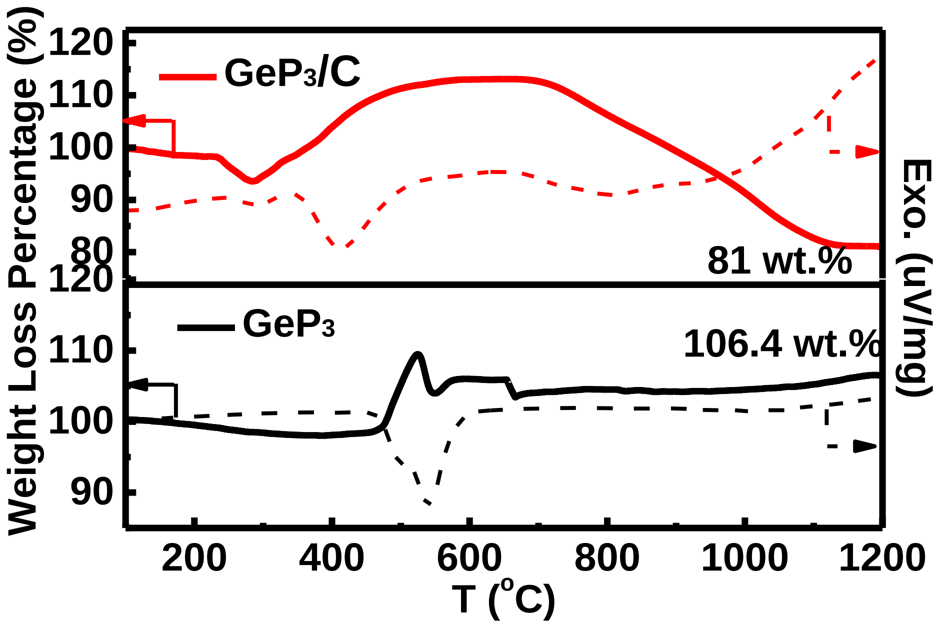

Fig. S8 TG and DSC curves of GeP_3 and GeP_3/C obtained by heating the samples in air atmosphere.

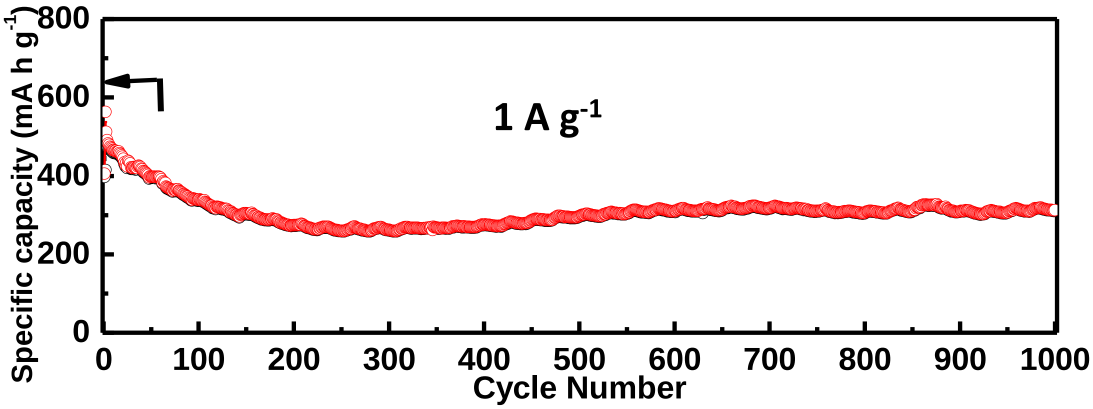

Fig. S9 Long-term cycling performance of GeP_3/C at a current density of 1 A g^{-1} (the specific capacity was calculated based on the whole electrode)

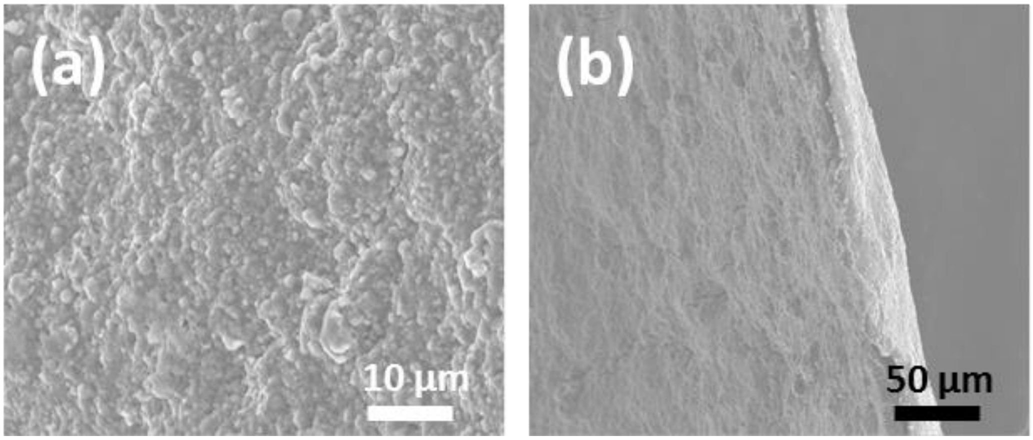

Fig. S10 SEM images of GeP_3/C after 30 cycles at a current density of 0.1 A g^{-1}. (a) Top side, and (b) cross section.

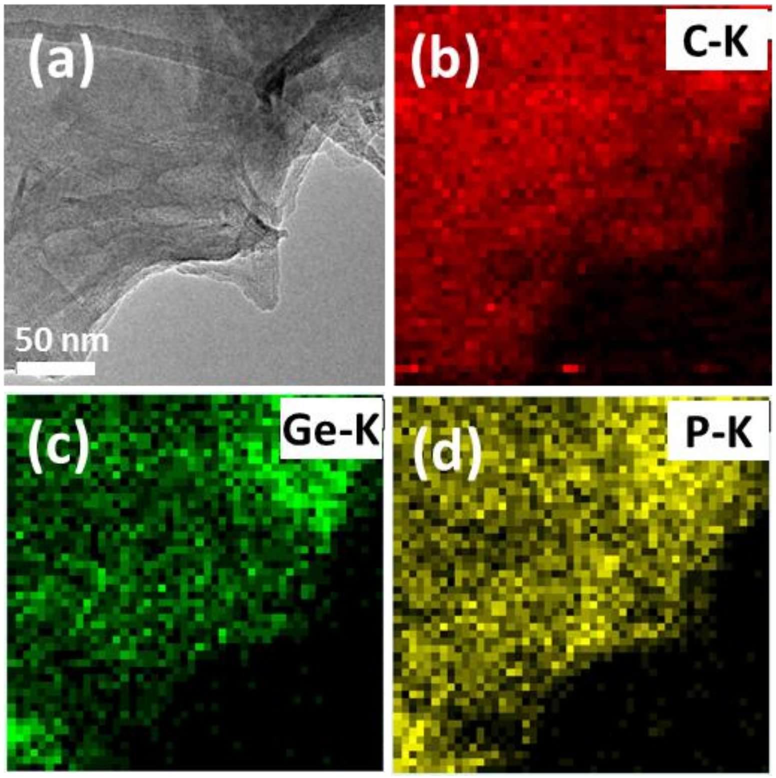

Fig. S11 TEM image and corresponding elemental mappings of GeP_3/C after 30 cycles at a current density of 0.1 A g^{-1}.
